# A Simple Method of Aspirating the Bladder

**Published:** 1875-10

**Authors:** Louis A. La Garde


A Simple Method of Aspirating the Bladder.—*By Louis A. la Garde.*—On the 10th July, 1875, a Mexican teamster, thirty-four years of age, entered the Post Hospital, with retention of urine of thirty-six hours' standing resulting from organic stricture of the urethra. He was put on opium, and given hip-baths frequently during the first six hours of his admission. Catheterization was attempted, but failed. He refused to submit to external perineal urethrotomy, which was urged as the only means of relief in the absence of any of the modern instruments for the relief of both the stricture and retention. Having no aspirator, and the opium and hip-bath treatment having failed, the case resolved itself into one of rectal or suprapubic puncture of the bladder. A happy idea occurred to me, which I received from my friend, Professor Griffith, of Kansas City, in a conversation last winter. I contrived an aspirator by means of a vacuum created in a quart bottle, in which was poured ℥ss. of chloroform, allowed to evaporate by heat in a water-bath near the boiling point. While the last of the chloroform was evaporating, the bottle was stopped with a tight-fitting cork, through which a canula was tightly adjusted. I attached a piece of India-rubber tubing, twelve inches in length, (from a Davidson syringe), to the end of the canula, and fixed a hypodermic needle in the other end of the tube. Having everything ready, I thrust the needle into the bladder, two inches above the pubes, in the linea alba. From the fact that the bottle was still warm, and the remaining chloroform, in a state of vapor, occupied the space in the vacuum, the urine did not flow for perhaps ten minutes; but as soon as the condensation of vapor commenced to take place, the urine was seen to come through the canula, at first drop by drop, and then into an uninterrupted stream. It continued to flow thus until the bottle lacked not more than two cubic inches of being full. The amount of urine removed measured two pints.

The morning following the operation the patient passed urine through the urethra, and later on in the day his condition was so much improved that he considered himself well, and insisted on leaving the hospital.—*The Medical Record.*

